# Clustering of cardiometabolic risk factors in sub-Saharan Africans

**DOI:** 10.11604/pamj.2021.38.112.28180

**Published:** 2021-02-03

**Authors:** Jean Jacques Noubiap

**Affiliations:** 1 Centre for Heart Rhythm Disorders, University of Adelaide and Royal Adelaide Hospital, Adelaide, Australia

**Keywords:** Dyslipidemia, diabetes, hypertension, cardiovascular disease, Africa

## Editorial

Dyslipidemia, defined as abnormalities in circulating concentrations of total cholesterol, low-density lipoprotein (LDL) cholesterol, high-density lipoprotein (HDL) cholesterol, and triglycerides [1], is a major risk factor for cardiovascular disease (CVD). It is associated with heightened risk of stroke, myocardial infarction, and is a major contributor to global disability and mortality. According to the global burden of disease study, in 2019, high LDL cholesterol caused 4.4 million deaths (7.8% of total deaths) and 98.6 million disability-adjusted life-years (DALYs) (3.9% of total DALYs) globally, representing the fifth and eighth risk factor for mortality and disability, respectively [2].

Sub-Saharan Africa is one of the regions with the sharpest increase in the burden of CVD [3]. It is estimated that CVD will overtake infectious diseases as the leading cause of death by 2030 in the region [4]. This epidemiological transition is driven by high rates of undiagnosed and uncontrolled cardiometabolic risk factors such as hypertension, diabetes, obesity and dyslipidemia [4]. Until recently, the burden of dyslipidemia had remained largely neglected in sub-Saharan Africa. A systematic review and meta-analysis published two years ago provided the first comprehensive estimates of dyslipidemia prevalence among adults living in Africa based on data from 181 studies involving 309,207 individuals [5]. In the general population, the prevalence of dyslipidemia was 37.4% for low HDL cholesterol (< 1.0 mmol/L), 28.6% for high LDL cholesterol (≥ 3.3 mmol/L), 25.5% for high total cholesterol (≥ 5.2 mmol/L), and 17.0% for high triglyceride (≥ 1.7 mmol/L). In people with diabetes and hypertension, the prevalence was respectively 42.1% and 39.4% for low HDL cholesterol, 50.1% and 32.7% for high LDL cholesterol, 34.4% and 38.0% for high total cholesterol, and 35.5% and 22.2% for high triglyceride [5].

The study by Fausto Ciccacci and colleagues published in this volume of the Pan African Medical Journal represents a relevant contribution to the epidemiology of dyslipidemia in Africa, and specifically in Mozambique [6]. The authors estimated the prevalence of high total cholesterol (≥ 5.2 mmol/L) in a group of 885 patients with hypertension, diabetes or both, recruited in an urban health facility in Maputo, Mozambique. They found that high total cholesterol was present in 46.3% of the total population, 46.9% of patients with hypertension, 36.7% of those with diabetes, and 52.8% of those with both hypertension and diabetes. Furthermore, high total cholesterol was associated with female sex (77% increased risk) and a body mass index ≥ 25 kg/m^2^ (50% increased risk) [6].

The high prevalence rates of dyslipidemia in patients with hypertension and diabetes found in this study and in other clinical settings in sub-Saharan Africa have important implications [5]. Dyslipidemia should systematically be investigated as part of the comprehensive cardiovascular risk assessment of patients with hypertension and diabetes, and treated appropriately. This is crucial, considering the impact of dyslipidemia on atherosclerotic CVD in sub-Saharan African populations. For instance, in the large Stroke Investigative Research and Educational Network (SIREN) study conducted in Nigeria and Ghana, dyslipidemia had the second greatest population attributable risk for stroke (36%) after hypertension [7]. Definitive evidence exists that effective treatment of dyslipidemia, with statins for instance, markedly reduces CVD morbidity and premature mortality [8]. Unfortunately, studies have shown that in sub-Saharan African settings, the prescription and the use of statins are low among patients who need it, such as patients with hypertension, diabetes, or established CVD [9,10]. For example, a study in Ghana revealed that only 1 in 6 individuals with type 2 diabetes without CVD and 1 in 7 stroke survivors were prescribed statins [9]. Another study in South Africa showed that 1 in 2 patients on lipid lowering medications did not reach target LDL cholesterol levels [11], mainly due to underdosing [12]. Improved prescription and access to statin and other preventive medications for CVD risk reduction is urgently needed in these settings.

The Prospective Urban Rural Epidemiology (PURE) study has shown that preventive medications including aspirin, β blockers, angiotensin-converting enzyme inhibitors, and statins are unavailable and unaffordable for a large proportion of communities and households in low-income countries [13], and that lower availability and affordability of such essential CVD medications are associated with higher risk of major adverse cardiovascular events and mortality [14]. To curb the rising burden of CVD in sub-Saharan Africa, governments and health organizations should devote special efforts to scaling up access to essential CVD medications using key interventions such as differential pricing, bulk purchasing, and use of low-cost generics. Additional measures include training healthcare providers on primary and secondary prevention recommendations through academic detailing, formularies and guidelines, and task shifting from physicians to nurses.

Because hypertension and dyslipidemia frequently co-exist [5], a fixed-dose combination therapy (polypill strategy) has been proposed as an approach to reduce the burden of CVD, especially in low-income and middle-income countries. Indeed, the effectiveness of polypills comprising statins, multiple blood pressure-lowering medications, with or without aspirin for primary and secondary prevention of CVD was demonstrated in some recent large randomized controlled trials [15-17]. Polypills were also shown to be associated with high medication adherence and low number of adverse events [15]. Interestingly, a modeling study showed that polypill would be cost-effective compared with current care for secondary prevention of atherosclerotic CVD in China, India, Mexico, Nigeria, and South Africa [18].

Finally, despite an increasing attention to dyslipidemia in sub-Saharan Africa in the recent years, there is still a huge knowledge gap regarding the determinants and adverse effects of this complex condition in the region. Efforts are particularly needed to expand our knowledge on the molecular basis of dyslipidemias in Africans [19]. Large-scale population studies are needed at national and regional levels to better understand the complex interplay between genetic, behavioral, environmental, social and cardiometabolic risk factors including dyslipidemia and their impact on cardiovascular health outcomes. Collaborative efforts such as the AWI-Gen and SIREN studies within the Cardiovascular Working Group of the Human Heredity and Health in Africa (H3Africa) Consortium should be multiplied on the continent [20,21].

**Disclosures:** Dr Noubiap is supported by a Postgraduate Scholarship from the University of Adelaide.
